# The influence of therapist actions, strategies and qualities on change processes in psychological therapies for eating disorders: grounded theory analysis

**DOI:** 10.1186/s40337-026-01713-8

**Published:** 2026-07-18

**Authors:** Jasmin Langdon-Daly, Annanya Goenka

**Affiliations:** Department of Psychology, University of Bath, Bath, UK

**Keywords:** Eating disorders, Psychological therapies, Therapist factors, Therapeutic process

## Abstract

**Background:**

Therapist actions, qualities and strategies are a potentially modifiable factor influencing outcome in psychological therapies for eating disorders. This study aims to hear the perspectives of those with lived experience, to identify therapist actions and behaviours which were helpful or unhelpful in change and recovery, and to construct a theory of the processes by which these therapist actions might impact on recovery.

**Methods:**

Eighteen participants who had undergone various psychological therapies for EDs took part in semi-structured interviews. They described helpful and unhelpful therapist behaviours, their emotional and cognitive impacts, and how these influenced recovery. Using a constructivist grounded theory approach, data were analysed through initial and focused coding, comparative methods, theoretical sampling, and memo-writing to develop a theoretical model.

**Results:**

Participants described two interacting major categories of therapist actions, strategies and qualities: therapist ‘style and stance’ along with ‘tasks of therapy’. These were described as having some direct pathways of influence on ‘change processes and recovery’, but also acted through three core categories of interacting intervening processes: ‘the person seen heard and cared for’, ‘trust and openness to therapy’ and ‘turning towards change’. These processes are the core of the model, acting to catalyse (or block) change.

**Conclusions:**

Elements of therapist style and the tasks of therapy influence recovery both directly and through key intervening processes: feeling seen, heard and cared for; developing trust and openness to therapy; and turning towards change. The constructed model and categories are considered in light of psychotherapy theory. The identified aspects of therapist style and tasks are relevant to clinicians and can inform the co-production of guidance for future use in training and delivery of ED therapies.

## Abstract (plain language summary)

Therapists play an important role in helping people recover from eating disorders, but we still know relatively little about *which* therapist behaviours are most helpful, and *how* they support change. This study explored these questions by listening to the experiences of people who had received psychological therapy for an eating disorder. Eighteen adults took part in in-depth interviews about what their therapists did that helped or hindered their recovery, how this made them feel and think, and how it influenced their willingness and ability to change. We analysed these interviews to develop a model explaining how therapist actions may affect recovery. Participants described two main aspects of therapy that mattered: the *therapist’s style and personal approach* (such as warmth, respect, and collaboration) and the *tasks of therapy* (what the therapist focused on). These influenced recovery both directly and by shaping three key experiences: *feeling seen*,* heard and cared for*; developing *trust and openness to therapy*; and *turning towards change.* These findings highlight therapist behaviours that may help create the conditions needed for recovery and can inform future training and delivery of eating disorder therapies.

## Background

A range of evidence-based psychological therapy approaches are recommended first line treatments for eating disorders (ED) [1]. However, individual responses vary, with only 30–50% achieving “good outcome” in trials [2, 3], and strikingly similar results across therapies compared. Treatment drop-out and relapse are common (greater than 25%) [4, 5].

Therapist characteristics and behaviours may represent a modifiable factor impacting on therapy outcomes. The outcome of any psychological therapy results from combination of ‘treatment-modality-specific’ factors and techniques, and those ‘common’ and relevant across all therapeutic approaches [6, 7]. Psychological therapies are not just a set of procedures or techniques, but an interactive, interpersonal process between therapist and patient. The person, actions, and relationship with, therapists plays an active and direct role in creating change, beyond any specific technique used [6]. Research exploring factors associated with outcomes in therapies for EDs has tended to focus on within-patient factors [8]. The role of therapist factors or behaviours has been neglected, despite this being part of the interaction which clinicians have most control over and ability to change.

Across mental health difficulties, there is evidence that ‘therapist effects’ (differences in outcomes for different therapists delivering same treatment) account for a significant proportion of variance in outcome, with larger impact for severe difficulties and longer treatments, (like ED therapies) [9]. A stronger therapeutic relationship is associated with better outcomes in ED therapies [3], but less is understood about therapist factors and behaviours which contribute to this relationship.

Ambivalence about change and recovery is a key element of many EDs which can influence course of therapy [10, 11]. People with EDs experience high distress, anxiety, threat, shame and self-criticism; particularly related to eating and change [12–15]. This can make tasks of therapy and recovery (requiring changing behaviour and confronting difficult emotions) particularly distressing and anxiety-provoking [16]. People with EDs report social difficulties and lower ‘safeness’ in relationships, and there is growing recognition of high prevalence of neurodivergence [13–15, 17–20] and the need for neuro-affirming care [21, 22]. Additionally, there may be clinician-client mismatch on conceptualisations and goals regarding recovery [23]. All of this could challenge development of the therapeutic relationship (increasing sensitivity to certain therapist behaviours), and maintain EDs.

Qualitative research has identified a range of therapist factors perceived as helpful or unhelpful. Many of these, such as showing empathy, being ‘supportive’, and avoiding judgemental criticism, appear similar to those that might be identified across a range of difficulties [24–31]. Others appear potentially more specific to EDs and treatments, such as attending to/ignoring needs beyond eating and weight, and offering nutritional guidance. The compelling accounts of people with lived experience in these papers offer important insight into the importance of certain therapist actions. They also frequently describe experiences at best unhelpful and at worst actively harmful to recovery. Manuals for specific ED therapies make suggestions about therapist roles, stance and behaviour [32–35]. These ideas tend to be based on clinical experience and expert opinion, and their influence on outcome has not been empirically explored independently of other modality-specific aspects of these treatment ‘packages’.

These papers and manuals do not explore mechanisms by which therapist behaviours, actions and factors might influence outcome. Some suggested mechanisms come from the field of psychotherapy process theory (not ED-specific). For example, the contextual model of psychotherapy suggests the outcome of any intervention occurs on three pathways: the ‘real relationship’ (direct change resulting from relational experience through evolved socio-emotional mechanisms), expectation, and inducement to make health-inducing behaviour changes [6]. The importance of these pathways, and specific therapist factors, qualities and strategies supporting or hindering them, is likely to vary between conditions, and may well be impacted by ambivalence and social/emotional changes seen in EDs [36].

### Study aims

To learn from the lived experiences of individuals who have undergone psychological therapies for EDs, to build an understanding of processes by which particular therapist actions, strategies and qualities support or block change and recovery.

## Methods

### Design

A constructivist grounded theory approach was chosen to centre, and build theory of inter- and intra-personal processes, from the accounts of individuals with lived experience [37]. The analytical approach aims to build theory empirically ‘bottom up’ from people’s accounts, before relating constructed theory back to relevant psychotherapeutic theory and ED treatment literature. The approach involved an iterative process of data collection (through intensive interviewing), and analysis. Credibility, resonance and usefulness were considered through triangulation with responses to a qualitative survey and review of existing qualitative literature.

### Participants

Eighteen people took part in interviews (sixteen women, one transgender man, one non-binary person). All participants were individuals who reported having experienced any kind of psychological therapy for any ED within the last three years (minimum four sessions with same therapist). Participants were all English speakers resident in UK. They were aged 18 and 54 (18–24 (7), 25–34 (3), 35–44 (5), 45–54 (3), with a range of self-reported ethnicity (Asian/Asian British (2), Mixed or multiple ethnicity (1) White/White British [15]). Fourteen participants described predominantly restrictive/anorexia nervosa (AN) presentations, two binge-purge/bulimia nervosa (BN) presentations, one a mixed picture of bingeing and restriction, and one atypical restrictive eating. Participants described a range of therapies including cognitive-behavioural therapy, MANTRA, family-based treatment, psychodynamic psychotherapy, compassion-focussed therapy, combined/integrative approaches, and unspecified ‘talking therapy’. All names presented with quotations are pseudonyms chosen by the participants.

Seventeen English-speaking individuals based in the UK, who identified themselves as having experienced psychological therapy for any kind of ED within last three years, completed the anonymous survey (for triangulation). Participants could complete both interview and survey.

Participants were volunteers who saw study advertisements shared through social media, university-based research advertising, two NHS ED clinical services, and other ED support organisations. Interview participants received a £20 shopping voucher. Survey participants were entered into a prize draw to win one of five vouchers. Recruitment continued alongside data analysis until theoretical sufficiency was considered achieved, with new data adequately accounted for by existing categories [38].

### Materials and procedure

Participants completed information sheets and consent forms on QuestionPro Survey Software [39] before arranging an interview time via email, or directly completing the online survey.

Individual interviews were conducted with JLD via Microsoft Teams videocall [40] or at University of Bath, ranging from 42 min to1hr5minutes, following a flexible topic guide. Participants responded to open-ended questions to describe helpful/unhelpful therapist actions, qualities or strategies, with follow-up questions exploring their impact on internal experience, and whether/how this impacted on making change and recovery. Interviews were recorded and transcribed verbatim by JLD & AG.

The online survey was developed through literature review of qualitative research to create a list of 18 potentially important helpful/unhelpful therapist factors in ED therapies. For each item (presentation order randomised), participants indicated if their therapist did this, listed example behaviours, and indicated if this was important to therapy outcome.

Research was approved by UK NHS Health Research Authority Research Ethics Committee (IRAS ID: 357647). The study was pre-registered on OSF. Topic guides and survey available https://osf.io/xh8yf. Data collected August-December 2025.

### Data analysis

Interview transcripts were analysed by JLD following procedures outlined by Charmaz [37] using NVIVO software [41]. Initial line-by-line coding of the first eight interviews led to development of a flexible focussed coding system - reviewing initial codes to highlight, sort, refine or collapse into tentative categories. Focussed codes were applied to data from subsequent interviews, with ongoing addition of new codes and refinement of coding system to encapsulate new meaning. Existing interview transcripts were revisited as part of the iterative process of constant comparison. Comparative methods (code to code, account to account, data to code and data to category), analytical memo-writing, and diagramming were used to construct and refine key categories from codes, and build into a process model.

### Researcher characteristics, reflexivity, and credibility of analysis

The constructivist stance acknowledges the active and intrinsically subjective interpretive role of researchers, requiring them to make explicit and interrogate the role of their assumptions, values and contexts in the constructed analysis. I (JLD) am a White British woman in my 30s, who is a clinical psychologist with 10 years clinical experience working with children and adults with EDs, using a broad range of psychological approaches (including nearly all those described by participants). My current role is clinical research and training. AG is an Indian woman in her 20s, currently an MSc clinical psychology student, without previous experience working with EDs or delivering psychological therapies. I maintained a reflexive journal throughout design and analysis, providing space to identify relevant contexts, assumptions and values and interrogate influence on analytic process.

To support credibility, procedures of investigator and method triangulation, and member checking, were applied. A subsample of five transcripts were independently coded by AG. Areas of similarity and difference in coding, and perceived potential categories, were discussed to shape and inform analysis. Developing categories and theory from analysis of interview data were considered alongside survey responses to ensure consistency with data gathered through this method. A draft findings summary was shared with consenting participants. Six returned comments which informed final analysis.

## Results

**Fig. 1:**
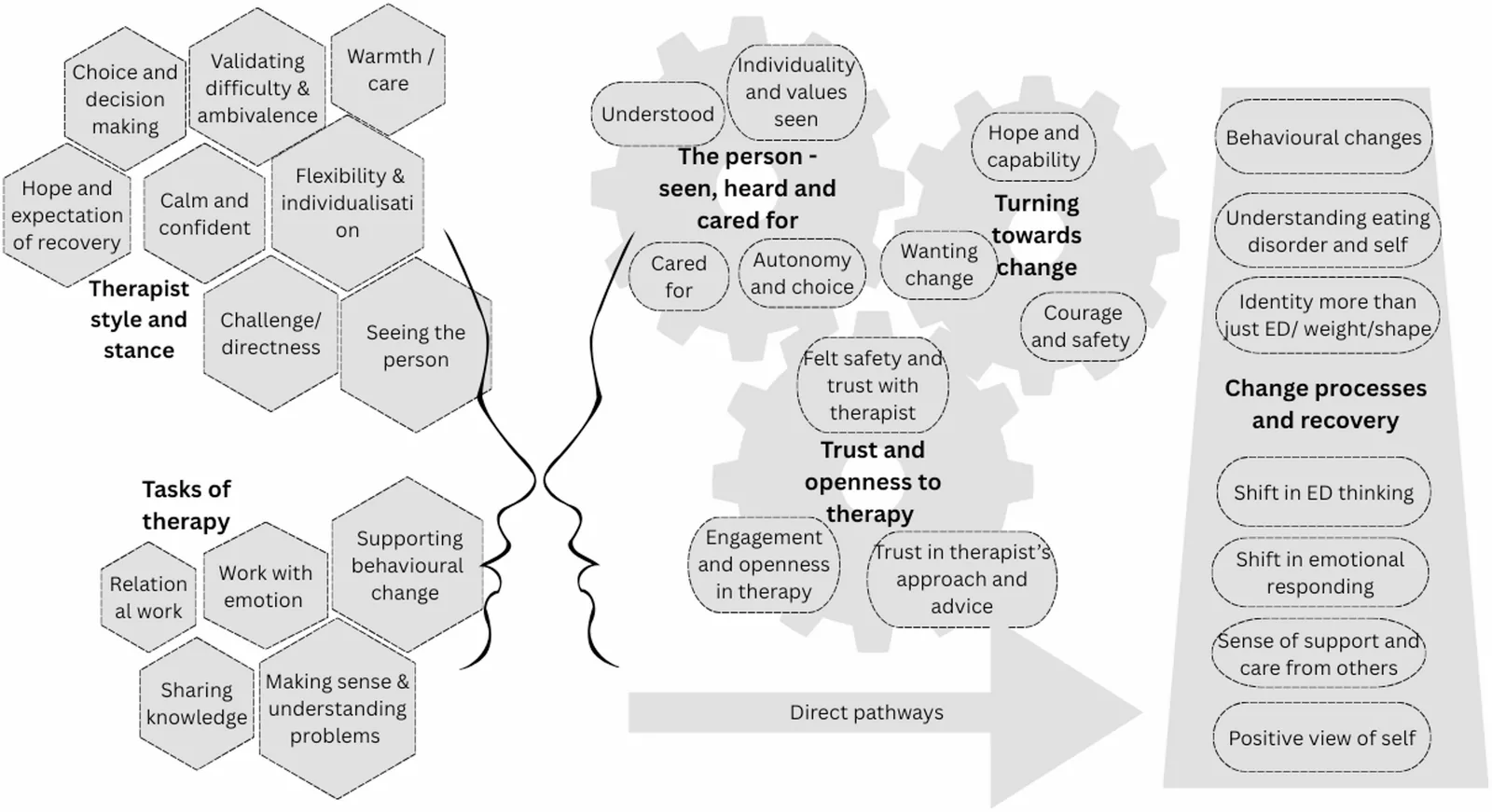
Model of therapist actions, and processes by which they influence, facilitate or block change and recovery in psychological therapies for eating disorders

An overview of the constructed theory (Fig. 1) is presented before describing each category. Participants described two major categories of therapist actions, strategies and qualities – aspects of therapist ‘style and stance’ along with ‘tasks of therapy’. These interacted, with stance and style of task delivery relevant to its reception and response. Therapist actions were described as having some direct pathways of influence on ‘change processes and recovery’, but also acted through three core categories of interacting intervening processes: ‘the person seen heard and cared for’, ‘trust and openness to therapy’ and ‘turning towards change’. These three processes are the core of this grounded theory, acting to catalyse (or block) change.

### Therapist style and stance

This category describes aspects of therapists’ interpersonal style and style of delivering interventions (8 subcategories); the ‘how’ rather than ‘what’ of therapy. A style incorporating these elements facilitates processes of change, and allows the person to benefit from ‘tasks of therapy’. Where helpful elements are absent, or the opposite is described, participants frequently described no change, disengagement, giving up, anger and even harm. Some subcategories appeared central in high numbers of accounts, whereas others were less frequently present (see Table 1 for example quotes for each subcategory).

**Table 1:** Therapist style and stance subcategories with example quotes

*Seeing the person* *Helpful/present* *Unhelpful / absent*	*So she knew that I wasn’t happy with my career, and then she knew I wanted to do something more meaningful, which I can help people with. So, for example, I wanted to teach yoga. So she noticed that, and then she encouraged me to do that.” - Eve* *“Assuming you don’t want to get better is a big one. Even if you’ve been sneaky and gone along with the eating disorder, it’s not out of enjoyment. It feels safe or like a means of survival in that moment. It’s not a fun time to keep going.” - Joanna*
Flexibility and individualisation *Helpful/ present* *Unhelpful/ absent*	*“Well*,* I suppose things like chronic pain and me saying I find it easier to communicate in this way*,* not in the way that’s usual or expected or traditional. She didn’t question that. She just trusted me that that would help me and then did those things. So it makes me feel like she was receptive. So I wasn’t going to be ignored.”* - Ashley *“So I really felt that body image wasn’t a concern for me at that point. I kept telling her that*,* and at the start she said*,* “OK*,* well*,* it’s part of the programme*,* it’s what we have to do.” -* Amelia
Challenge/ directness	“*And so I think whilst I didn’t like being called out*,* she did it in a way that was kind of like*,* “this is what you want”. …It’s that you have to be cruel to be kind*,* almost. And it wasn’t cruel*,* you know*,* it certainly wasn’t. But she knew it was going to be uncomfortable*,* because recovery is uncomfortable.” -* Florence
Warmth and care	*“Yeah*,* it was just*,* I guess*,* going out of her way and going off like the norm sessions. Like*,* this on the timetable so we have to do this. But actually just doing something out of that to make a difference to my day” -* Jenna
Validating difficulty and ambivalence	*“She never*,* ever belittled anything that I was feeling or finding difficult. She didn’t understand it from a lived point of view*,* but would always recognise that for me and for other people that she works with the strength of the feelings is very high and real.”* - Tulip
Offering choice and shared decision making	*“It felt like I could almost pick and choose*,* the modules that we did in MANTRA*,* or the times that we had appointments. It was*,* I guess without it being like ‘it’s all down to you’*,* it was all down to me. And I really liked that. It was*,* it was my problem. It’s my eating disorder. It’s my choice to recover from it.”* - Anya
Hope and expectation of recovery	*“Their exact words were ‘you seem like you’re living like a fairly good quality of life at a low weight*,* so maybe we should just shift the focus trying to fully weight restore or something*,* to just managing things where they are’. And I found that*,* again*,* really hard to hear*,* because I had been trying to say ‘I want to keep making progress’. But then for someone else to say to me ‘How about we just stop trying’. It felt like they were giving up*.” - Molly
Calm and confident	“*Not reacting with*,* “Oh my God*,* that’s awful*,*” or recoiling in disgust or fear*,* but saying*,* “OK*,* that sounds tricky. Let’s discuss it.” -* Rosie

#### Seeing the person

This subcategory is central to many accounts, and underlies several others (e.g. individualisation, directness/challenge). Participants spoke of intensely valuing therapist actions demonstrating seeing them as an individual human with interests, values, goals and strengths. Example behaviours included asking about, remembering and referring back to aspects of people’s lives, and noticing and commenting on values and strengths perceived. Engaging at a more surface level e.g. asking about hobbies, or appearing to ‘miss’ the individuality of the person was described as unhelpful.

Participants spoke about unhelpful experiences of therapists making assumptions about them as ‘a person with ED’. This was particularly noted as unhelpful when therapists expressed lack of trust in the person because of ED. Participants named some approaches to weighing/weight in treatment – for example as the first part of any session, key topic of conversation, or deciding factor in treatment decisions, as examples of therapists not seeing them as a person or reducing them to a number.

Several participants commented on the importance of therapists seeing and understanding the potential role of their culture in their life experience and ED.

#### Flexibility and individualisation

This subcategory was similarly central. Many participants spoke of unhelpful therapist actions demonstrating a rigid, inflexible and non-individualised approach. Several described therapists explicitly or apparently sticking rigidly to a manual or ticklist, rather than responding flexibly to important aspects of what this individual person was bringing or needing. Participants also described rigid meal plans or behavioural instructions, which were not tailored to the life circumstances, preferences or needs of the person.

Many examples were given of helpful therapists taking an individualised approach to treatment, which addressed things that needed to be addressed to support change for that individual, and/or included adaptations (for example practicalities or communication) to accommodate needs and preferences. Flexibility was valued in choice of tasks and also in pacing.

Many participants noted that ‘everyone is different’ and found it helpful when therapists were responsive to their feedback on relevance/ comfort of different approaches and tasks. In contrast, several spoke of therapists not listening or ignoring their feedback or requests. Some described experiences of therapists offering the same treatment approach for several years without alteration, despite no improvement.

Several noted helpfulness of having the option of extending/no fixed numbers of sessions, if more time was needed. Where comorbidity was present, flexibility to include this in the work was described as highly helpful, in contrast to therapists who excluded working with comorbid emotional difficulties or risk.

#### Challenge/ directness

Many participants described the importance and value of therapists being direct in questioning and challenging ED thinking and behaviour, and in naming and highlighting the need for change. This challenge and directness was particularly experienced as helpful when it was framed with compassion and understanding, and involved highlighting ways the ED was getting in the way of an individual’s goals or values. This approach was described as more helpful than use of ‘threats’ of ‘what will happen if they don’t change’, or where challenge was linked with rigidity or distrust. Failure to challenge, being taken in by or colluding with the ED, or simply ignoring it, was named by some as unhelpful.

#### Warmth/ care

Participants described valuing therapists showing emotional warmth and friendliness, visibly listening, and expressing liking and positive regard for them. They noted examples of actions where therapists had gone beyond the ‘required’ or expected, gestures showing they had been thinking about someone, and sticking with someone through crises or difficult life situations, as examples of demonstrating care. Several noted therapists sharing small details of their own lives as an example of helpful behaviour, expressing liking or trust. Examples of uncaring behaviour described included not doing things agreed, missing appointments, telling someone they are not a priority, or not trying to rebuild relationships after rupture.

#### Validating difficulty and ambivalence

Several participants highlighted the importance of therapist’s taking the ED seriously, taking concerns seriously, and validating worries and distress. Being non-judgemental and understanding when people had struggled to make change, experienced high distress or were expressing ambivalence, was named as helpful.

#### Offering choice and shared decision making

Many participants named therapist actions around choice and decision making as relevant. Participants described it as very helpful when therapists prioritised their wishes and needs, highlighted and supported their choice and autonomy, set goals with them, discussed and made decisions together. Offering choices, rather than ‘telling what to do’ was helpful, although it was important that these were genuine (options that were actually available). Even in situations where choice was limited, transparency was named as important, including explaining reasons behind treatment suggestions or decisions, involvement in decisions and conversations about them, and not ‘going behind someone’s back’.

#### Hope and expectation of recovery

Expressing confidence that recovery is possible, and that this person is capable, was named by several participants as important. Talking about future life without ED, connecting with or sharing stories of recovered others, and staying committed even through difficulty, were seen as expressions of belief in this. Several participants shared examples of unhelpful behaviours such as telling someone they were likely unable to recover, setting goals short of full clinical recovery, and talking extensively about how long someone has been in treatment.

#### Calm and confident

Several participants named helpfulness of therapists remaining calm, particularly in face of high distress or conversations about risk. Appearing flustered, anxious or unable to manage distress were described as unhelpful and uncontaining. Apparent confidence and explicit or implicit modelling that change is safe were helpful.

### Tasks of therapy

The specific tasks of therapy and their relative importance varied between individuals and therapeutic approaches. There were some common factors arising in accounts of multiple different therapy types. Some subcategories appeared more consistently across multiple accounts, while others appeared less often and seemed more or less important depending on the nature and formulation of maintaining factors for each individual’s ED. These tasks are described only briefly here, as they are covered extensively in broader modality-specific literature (see Table 2 for example quotes for each subcategory).

**Table 2:** Tasks of therapy subcategories with example quotes

Understanding and making sense of the problem	*“Like I’d say something, ‘Oh yeah, that happened. I don’t think it was important.’ And they’d go, ‘No, but how did it actually make you feel? What happened after that point in time?’* “ - Anya
Supporting behavioural change	“*Something helpful that comes in mind*,* is when I’ve been set pre/ between session tasks. Because like the test and try type of approach to adaptation. And when the therapist has suggested as a practical task*,* why don’t you try that*,* then report back*,* I’ve always found that approach really helpful.”* - Indra
Working with emotion	*“I do think that like exposing*,* getting the emotions kind of out and expressing them and feeling them was really helpful. And I do think that there was a lot going on when I was keeping it all inside and trying to numb it with various eating disorder behaviours.”* - Maeve
Sharing knowledge	*“So in theory*,* the plan would be that you eat three meals a day and you can have two snacks*,* a snack in between both…But you want more information than that to get you through. Most people who are therapists*,* I’ve found*,* haven’t been able to take you any further than that piece of information.”* - Dixie
Relational work	*“She also told me that my partner could join some sessions*,* because he was also very lost” -* May

#### Understanding and making sense of the problem

Understanding the problem was important for tailoring interventions, but also for helping the person to make sense and develop understanding of their own difficulties. Asking detailed questions about an individual’s thoughts, emotions, behaviours and experiences, and helping to understand links between them was important. Several participants described experiences where this was missed, giving examples of therapists making quick assumptions, or sharing theories of ‘all people with EDs’.

#### Supporting behavioural change

Therapists proactively supporting behavioural change was a key task described by many participants. Example actions included setting concrete behavioural tasks, plans and goals, talking through and problem-solving change, and even doing change tasks alongside someone. Behavioural changes supported by therapists included eating changes but also encouraging change in hobbies, working life, emotional responding or relationships. Several named therapists encouraging them to start with small changes, framing change as an ‘experiment’, and noticing and acknowledging changes made, as helpful. Suggesting behavioural changes without consideration of how they might look for that individual/their life circumstances was described as unhelpful and unlikely to be acted upon.

#### Working with emotion

Some participants named therapists focussing on understanding, acknowledging, and responding differently to emotion as crucial. This was particularly apparent in accounts of individuals with binge-eating type difficulties, but also named by several with restrictive difficulties (often in relation to reducing emotional avoidance). Several participants gave unhelpful examples of therapy where emotional content had been ignored or not responded to.

#### Sharing knowledge

Some participants noted helpfulness of therapists having and sharing factual information and strategies about nutrition, ED, physical health, and emotion regulation. Sharing dated or irrelevant information, or seeming to lack specialist knowledge was unhelpful. Sharing information was helpful when it done calmly and without claiming to ‘know everything’.

#### Relational work

Some participants described helpful examples of therapists discussing relationships, and involving family and loved ones in treatment. Involving others was described as unhelpful where it was ‘forced’, where families were overinvolved, or where family members were being talked toinstead of the person.

### The person seen, heard and cared for

Participants’ descriptions of internal responses to therapists actions highlighted a key facilitating or blocking process of feeling seen, heard, respected and cared about on a human level. These cognitive-emotional experiences played a role in engagement and trust, supported hope and confidence, and also acted directly on processes of change and recovery. This process consisted of four interacting and overlapping subcategories (see Table 3 for example quotes for each subcategory).

**Table 3:** The person seen heard and cared for subcategories with example quotes

Individuality and values seen	*“That I can still feel like someone’s noticing that I’m not just, you know, patient in bedroom #2, but I’d actually had a background. And I think that was quite nice. It just made me feel like, OK, I haven’t quite lost all of it.”* - Rose
Understood	*“I just felt like they were dismissing my point of view. And I feel like from what they were thinking*,* from their point of view*,* I feel like that didn’t really apply to me. So I think I thought at the time what they weren’t saying wasn’t useful.* - June
Cared for	“*But I knew that they only cared about me when I was skinny and I liked feeling cared about.” -* Anya
Autonomy and choice	*“I think if I’d been told I had to do it*,* I’m not sure if I would have resented it a bit. And been reluctant and more afraid of doing it. But I suppose because I it felt like I was giving permission for it to happen*,* somehow just coming from a different place of me saying ‘Yeah*,* I’m ready to do it’ or ‘I think I’d like to do it this way’*,* somehow made it easier.” -* Tulip

#### Individuality and values seen

A key experience was a sense of having their individuality and values seen (by the therapist), and feeling seen as a person, not just a patient. Several participants named this as a key process in their recovery – allowing them to notice, trust and develop their own broader identity away from ED, and develop confidence in themselves. Where this was lacking, participants described experiences of feeling uncared for, assumptions being made, and missed elements of treatment and recovery.

#### Understood

Many described a sense that their therapist understood them, the things they were saying, and the nature of their difficulties. There was a sense of compassionate and validating understanding – that their struggles were understandable and taken seriously. An equal number of participants described a view that their therapist didn’t understand them, their difficulties, their goals, their values or their distress. These participants tended to describe poor therapy outcomes.

#### Cared for

Participants feeling cared for and experiencing emotional warmth from therapists was crucial to feeling safety and trust with them. This sense of being cared for by therapists contributed to a broader change process of believing and trusting that they would be cared about by others without their ED, along with a more positive sense of self overall. Feeling cared about by therapists was particularly important to some, with feeling uncared for leading to hurt, anger, upset, disengagement, or even escalation in ED behaviour.

#### Autonomy and choice

A sense of having autonomy and choice about treatment and recovery, being respected and in control, was crucial to several accounts of engagement and confidence to make change. Other participants talked about feeling patronised, ‘done to’, or even coerced or deceived, commonly leading to disengagement, mistrust and anger.

### Trust and openness to therapy

Participants needed to be willing to approach, trust, be open and engage in therapy, for therapeutic change to occur. This was a key process by which therapists’ actions influenced outcome (see Table 4 for subcategories with example quotes).

**Table 4:** Trust and openness to therapy subcategories with example quotes

Felt safety and trust with therapist	*“Overall I find it difficult to trust people in services. It doesn’t always feel like they want what you want. Not that what I want is always good — but sometimes it feels like you’re not heard, or you ask for support and get something completely different. After being in hospital, I found it harder to be honest because I felt like my honesty was sometimes used against me. So feeling like you have a real connection with someone — because you talk about something other than your eating disorder — helps build trust. “* - Lulu
Trust in therapist approach and advice	*“And if someone who I could trust was kind of encouraging me to do these things*,* saying like this is what will ultimately help you*,* it just made me feel like maybe it is worth giving it a go.”* - Molly
Engagement and openness in therapy	*“It feels frustrating. You don’t want to make them proud or tell them positive things. It brings out the rebellious side of the eating disorder: “You don’t believe in me*,* so why should I believe what you’re telling me?” -* Joanna

#### Felt safety and trust with therapist

Many participants described feeling they could trust therapists as a safe person to be open and vulnerable with. This sense of trust emerged from therapists appearing calm, confident, warm, understanding and caring, being transparent and involving them in decisions, and appearing to trust the person. Felt safety with therapists was helpful for emotion regulation, facilitating clear thinking and behavioural change (for example through the actual or internalised presence of the safe therapist). A significant number of participants described feeling unable or unsafe to trust therapists, with poor outcomes universal in this group. Once lost, trust was described as almost impossible to rebuild.

#### Trust in therapist approach and advice

Alongside trust in the therapist as a person, participants described the importance of feeling they could trust the therapist’s approach or advice as safe to follow and likely to help. This was crucial to feeling hopeful about recovery and trialling suggested changes. Therapists or approaches being seen as unable to help frequently led to hopelessness and giving up.

#### Engagement and openness in therapy

Trust, hope, autonomy and feeling understood and validated, were commonly linked by participants with being willing and able to engage fully in therapy, be open in therapeutic conversations, and take actions based on therapeutic suggestions. Experiences of this lacking, disengagement, ‘checking out’, giving up, and anger towards therapists, were common in accounts of therapy experiences ending in no change, deterioration or early termination.

### Turning towards change

Approach towards change was a key facilitator of change itself. Participants described therapist actions, trust, and being seen and believed in as a person, leading to experiences of resolving ambivalence, courage/reduced fear of change, and a sense they were capable and change was possible. These experiences combined to drive and support the behavioural changes required for recovery (see Table 5 for subcategories with example quotes).

**Table 5:** Turning towards change subcategories with example quotes

Wanting change	*“I think it made me do a lot more reflection on myself, and be more a lot more transparent as to, what are my behaviours that I’m doing still that I don’t really want to be doing when I’m 80 years old? And if I’m still doing them now, then what’s going to change if I don’t stop it?”* - Florence
Hope and capability	*“You felt OK so then*,* yeah*,* we can sort this out. It’s not just all in - I know it’s all in my brain- but*,* you know*,* it can be treated. You can*,* you can help yourself. It can…It was all positive.” -* Poppy
Courage and safety	*“It feels like a safety net*,* so I’m more likely to walk along the tightrope because there’s a safety net”*- Rosie

#### Wanting change

Therapist actions such as directness and challenge, sharing information, highlighting individual values and goals, and validating ambivalence, were described as contributing to shifts in their sense of need and desire for change, awareness of the harms and downsides of ED, and there being ‘more to life than ED’. Participants described realising they needed to do the work of recovery and take action, and of wanting to make a ‘full’ recovery. Where therapists were experienced as not seeing the individual, not caring, colluding with ED, or expressing lack of hope, participants described increased thoughts that change is not necessary, reduced willingness to give up ED (for example as a means of getting care), or giving up.

#### Hope and capability

A sense that recovery is possible, that therapy and work is likely to result in a good outcome, was key to engagement and willingness to do the work of change. Participants also described the importance of confidence in their own capability to make change. This was largely dependent on perception of the therapist’s own belief in their abilities and expectation of a good outcome, along with their faith in the therapist and their approach.

#### Courage and safety

Making change and recovery were described by several participants as stressful, difficult and feared. Trust, felt safety, a sense of being supported and cared for by a confident therapist, and belief in therapist’s approach, were described as helpful in increasing sense of safety in, and courage for, change.

### Change processes and recovery

Participants described a range of changes processes and aspects of recovery (7 subcategories). These are listed but not described in detail here, as ED recovery aspects have been extensively described in other literature [42–45]. Table 6 lists the subcategories with example quotes.

**Table 6:** Change processes and recovery subcategories with example quotes

Increased understanding of self and ED	*“It was really helpful, because I finally had a way to visualise these characters. When we were doing a lot of the cognitive restructuring and questioning, I could think, “OK, this thought isn’t part of me, it’s the character.” One of the characters was like a really fierce school-teacher type, and I’d think, “Oh, it’s the fierce headteacher – she’s telling me to do this.” - Amelia*
Behavioural change	*“And you know*,* certain experimenting with challenging things I really didn’t like and was very much kind of ambivalent about doing. But I think I was encouraged to be willing to at least give it a go and I think I am fortunate at this point*,* to have a bit of hindsight from past experiences that actually*,* I do know it does get a bit easier.”* - Florence
Shift in ED thinking	“*But I did then just kind of notice that I’d get a disordered thought and I kind of just go ‘It’s not Wednesday*,* it’s not 2:00 PM*,* don’t*,* this is this is none of my business. Nothing to do with me. Go away.’ And I’d be like aware that the thought had happened*,* but it wasn’t any of my business. It was not something I’m interested in dealing with or developing or anything at that point.”* - Anya
Identity broader than ED, weight and shape	“*It helps you realise there are things you can bring into your life and focus on*,* and that you can pour energy into. Whether rediscovering things from before the eating disorder or picking up new things*,* that’s important and puts therapy in good stead.” -* Joanna
More positive view of self	*“To have … a fellow human being who I held in high regard*,* affirm that I was in some way being successful in enacting something which was so important to me on a human level. It was kind of a beautiful*,* a wonderful*,* powerful moment of validation. It made me feel*,* there is hope in my humanity. There is hope for me as a person. I’m not a complete failure.”* - Indra
Shift in emotional responding	*“I think first of all*,* having recognised what those emotions are*,* it is easier to kind of also like pinpoint the emotions*,* and oh think maybe I’m not that*,* you know what I mean? Like it kind of divert or change the direction of thoughts*,* when I labelled or name those negative feelings or emotions.”* - Eve
Sense of support and care from others	*“Knowing there was support*,* that love and support were stronger than whatever the eating disorder was offering*,* helped. The thought that people around me wanted to help”* - Joanna

An *increased understanding of their ED and self*, including making sense of triggers and knowing how to respond and what to do to get and stay well, were central to accounts of positive outcomes, facilitating other processes of change.

*Behavioural change* both to eating-related behaviours, and broader life activities, relationships and responses to emotions, were crucial to many accounts of recovery and good outcomes. This was often accompanied by accounts of *shifts in ED thinking* (with ED concerns and clarity/confusion increasing or decreasing).

Several participants talked about shifts in their sense of self. In particular, an increased sense of their *own identity being broader than just ED*,* eating*,* weight or shape*, along with (for some) a generally more *positive and compassionate view of themselves.*

For some participants where emotions and responses to them were a key part of their ED, *shifts in emotional responding* was a key change processes influenced by therapist actions. Several participants described helpful shifts in their *sense of reliable support and care from others*, driven by feeling cared for by the therapist and having other loved ones more involved through treatment.

## Discussion

The research identifies therapist actions, qualities and strategies identified as helpful and unhelpful by people with lived experience across the range of psychological therapies for EDs, and develops a model of processes by which these could influence change and recovery. Therapist actions included both the tasks of therapy and the style used in delivering these. Key tasks were making sense of the eating disorder and related difficulties, and supporting behavioural change, alongside knowledge sharing and emotion or relationship work where required. Clinician behaviours that demonstrated to the client that they were seen as an individual person (with goals, values, and unique identity outside of their eating disorder), and taking an individualised flexible approach were central to most accounts. A style described as helpful requires balance of challenge/directness, validation of difficulty, warmth and care, supporting choice and decision making, and offering hope and confidence. These therapist actions are conceptualised as having some direct influence over processes of clinical change and recovery, but crucially also acting through three interacting catalysing/blocking processes– the person’s sense of being seen, heard and understood; trust, openness and engagement in therapy; and a cognitive-emotive process of turning towards (or away from) change.

The categories and model developed are consistent with aspects of ‘common factors’ theories such as the contextual model [6]—highlighting the role of transformative relational experience alongside behaviour change and hope/expectation. ‘The person seen heard and understood’ subcategories also bear similarity to theories of core human needs (autonomy, competence and identity [46]) or core emotional needs (safety, nurturance, acceptance, autonomy, competence, identity and freedom to express valid needs/ emotions).

The research is distinct in that the model is built ‘bottom up’ and grounded in individuals’ accounts of lived experience. There are also aspects of the model which feel specific to EDs and their treatments. In particular, EDs (particularly AN) are often described in relation to central experiences of ambivalence, overfocus on weight and shape, control, and inflexibility [10, 19]. It is striking that rigidity, control, and neglect of broader identities, emotions, and relationships were often described as replicated in unhelpful therapist actions and strategies [7].

The category ‘turning towards change’ highlights that ambivalence/ motivation in EDs should not be conceptualised simplistically as wanting change or not, but is a socio-emotive experience which also includes fear/courage and hope/capability. This understanding bears similarity to models of motivation and behaviour change, such as the transtheoretical model (including decisional balance and self-efficacy [47]), while also highlighting the importance of considering emotional experiences of fear of change, safety, and courage. A sense of felt safeness and trust, which allows for taking in and acting on ‘the message’ (similar to concepts of epistemic trust [48]), was described as relevant to managing this, as well as creating the circumstances for openness and engagement in therapy.

### Strengths and limitations

This research represents an attempt to hear from the voices of individuals with lived experience, and to contribute to the ‘helpful factors’ literature, but also to go beyond this to consider processes by which therapist factors actually influence change and therapy outcome. The complexity of the model may impact on its research utility, but represents an attempt to effectively theorise complex experiences, allowing space for nuance and individual difference. Further research would be needed to establish if these therapist behaviours are potential correlates of therapy outcome, and any empirical consideration of causality would be complex given the broad range of other interacting variables which would also impact outcome.

The findings (and analysis) are inherently subjective and rely on the recall and perspective of the research participants, as perceived by the researcher, rather than some objective observations of therapy sessions. The credibility and resonance of the analysis is supported by triangulation with other data sources and coders, and member checking. Conversations about draft analysis with clinicians and individuals with lived experience noted the resonance and usefulness of findings to their experience and practice, and have informed the final presented model. The participant group includes some diversity in presentation, therapy type, age, gender and ethnicity, but the group is undeniably UK-based, with the majority of therapy experiences occurring within UK healthcare contexts, and predominantly consists of White British women with restrictive EDs. Clinicians and researchers will need to consider the transferability of findings to their contexts and populations.

Throughout the paper, and when asking questions in interviews with participants, the terms ‘recovery’ and ‘positive change’ were used without specifying a definition. There is potential for different perspectives and definitions of recovery between individuals and groups (e.g. people with lived experience / clinicians [49]). This decision was made with intention to prioritise the perspectives and experiences of participants, allowing them to use their own internal definition of ‘recovery’ and ‘positive’, rather than imposing one. Since participants were not asked to share how they were defining positive change or recovery, the researcher or readers of the research have no way to confirm if participants were using a similar or different definition to themselves. This is a limitation when considering and applying the findings and model. *Future directions and clinical implications*.

Co-production work could be used to develop descriptions of helpful/unhelpful therapist behaviours into useable clinician guidance, checklists and observational tools allowing clinicians, supervisors, clinical trainers and researchers to observe the presence/absence of behaviours, or even discuss them with clients at reviews. These tools could be used in observational studies investigating hypothesised processes of change (e.g. interpersonal process recall), to explore associations between behaviours and therapeutic outcomes, and to consider applicability to diverse populations. Guidance and observational tools could be evaluated to establish whether they are useful in shaping therapist behaviours.

The findings could guide the delivery of psychological therapies for EDs. This is clearly a challenge - balancing different aspects of style and tasks of therapy, requiring clinicians to be ‘both/and’ rather than ‘either/or’, in the face of individual differences and contextual limitations. The high levels of clinical risk in ED work may potentially have an impact on clinical style (for example in increasing risk averse, rigid or controlling, weight focussed practice). The outcome of any therapy will of course also depend on the person in treatment, their difficulties and work in making change. While acknowledging this difficulty, this research could be useful for clinicians to reflect on their own practice and part in therapy outcome, and do what they can to deliver therapies in ways which support recovery and positive change, and minimise disengagement.

## Conclusions

This study explores the perspectives of individuals with lived experience on the role of therapist actions, strategies and interpersonal stance in shaping processes of change and recovery in psychological therapies for eating disorders. The constructed grounded theory model suggests that therapist style and the tasks of therapy influence recovery both directly and through key intervening processes: feeling seen, heard and cared for; developing trust and openness to therapy; and turning towards change. These findings emphasise that therapist behaviours actively shape engagement, hope and willingness to change in a population characterised by ambivalence and high distress. Attending carefully to how therapy is delivered, alongside what is delivered, may help optimise outcomes, reduce disengagement, and inform training and delivery of eating disorder treatments across therapeutic modalities.

## Data Availability

Survey and interview questions, anonymous transcripts of interviews and anonymised survey data are available on OSF [https://osf.io/xh8yf](https:/osf.io/xh8yf) .

## Abbreviations

ED: Eating disorder
